# Association of carvedilol with mortality in critically ill patients with sepsis: a retrospective propensity score-matched cohort study

**DOI:** 10.3389/fphar.2026.1796036

**Published:** 2026-09-21

**Authors:** Hua Xie, Chaohao Guan, Xiao Xu, Shufeng Liao, Junhua Yang, Lixia Yang, Zhizhi Yu, Yuejue Zhang, Bin Liu, Jun Wang, Yuzhan Kang, Jinlian Shao

**Affiliations:** 1 Department of Emergency, Zhujiang Hospital, Southern Medical University, Guangzhou, China; 2 Department of Neurotrauma and Neurocritical Care Medicine, Zhujiang Hospital, Southern Medical University, Guangzhou, China

**Keywords:** carvedilol, MIMIC-IV, mortality, propensity score matching, sepsis, β-blockers

## Abstract

**Objective:**

To evaluate the association between carvedilol use and mortality in patients with sepsis.

**Methods:**

All adult critically ill patients with sepsis were retrospectively identified from the MIMIC-IV database. Patients who received carvedilol between sepsis diagnosis and Intensive Care Unit(ICU) discharge were designated as the intervention group and were matched 1:1 with non-users using propensity score matching. The primary outcomes were 28-day and 365-day all-cause mortality, and the secondary outcome was in-hospital mortality. Kaplan–Meier survival curves and Cox proportional hazards regression models were used to examine the association between carvedilol and outcomes. Subgroup analyses and sensitivity analyses including time-dependent Cox regression were conducted to test robustness.

**Results:**

A total of 33,453 patients were included, of whom 1,084 received carvedilol. After propensity score matching, 1,082 pairs were successfully matched. Kaplan–Meier survival curves showed significant differences in survival probabilities between the two groups. After adjusting for confounders using multivariate Cox regression, the hazard ratios (95% confidence intervals) for carvedilol use on 28-day and 365-day all-cause mortality were 0.53 (0.43–0.66) and 0.66 (0.57–0.76), respectively. Furthermore, carvedilol use was associated with lower in-hospital mortality (OR = 0.49, 95% CI: 0.37–0.64; P < 0.001). Subgroup and sensitivity analyses supported the robustness of the findings.

**Conclusion:**

Carvedilol use during the period from sepsis diagnosis to ICU discharge was associated with reduced mortality in critically ill patients with sepsis. However, prospective studies are warranted to further guide the use of carvedilol in this population.

## Introduction

Sepsis is a life-threatening organ dysfunction caused by a dysregulated host response to infection (Rudd et al., 2020). It affects approximately 50 million people annually worldwide and is one of the most common causes of death (Evans et al., 2021). Conventional management of sepsis involves controlling the source of infection through antibiotics and supporting failing organs with fluid resuscitation, vasoactive agents, and mechanical organ support (Evans et al., 2021; Angus et al., 2013). However, this approach does not adequately address the dysregulated host immune response during sepsis; consequently, immune-based therapeutic strategies have been proposed (van der Poll et al., 2017).

Sustained sympathetic nervous system stimulation is an integral part of the physiological response to sepsis and can lead to downregulation of cardiac β-adrenergic receptors as well as tachycardia (Hollenberg and Singer, 2021). Hayase N. et al. demonstrated that tachycardia is an independent predictor of poor prognosis in septic patients (Hayase et al., 2016). Bucher M. et al. found that endotoxemia induces systemic downregulation of α1-adrenoceptors in all organs examined, accompanied by marked increases in tissue concentrations of interleukin-1β and tumor necrosis factor-α (Bucher et al., 2003). Carvedilol is a medication commonly used to treat cardiovascular diseases, including hypertension and chronic heart failure (Ruffolo et al., 1997); it acts by reducing sympathetic activity and controlling heart rate. This drug is a non-selective β-adrenergic receptor antagonist that also induces vasodilation by blocking vascular α1-adrenoceptors. However, recent studies suggest that the protective effects of carvedilol may extend beyond the cardiovascular system. Its unique pharmacological properties, such as antioxidant, anti-inflammatory, and anti-fibrotic actions (Ruffolo et al., 1997; Dandona et al., 2007), have attracted research interest in other disease areas. Administration of carvedilol to rats subjected to cecal ligation and puncture significantly reduced serum levels of IL-6 and TNF-α. Evidence indicates that adrenergic receptor blockers can improve outcomes in inflammatory conditions such as burns, surgery, and viral myocarditis, while also reducing the expression of inflammatory factors (Abdel Kawy, 2015).

In this context, we conducted a retrospective study to determine the association between the use of carvedilol and mortality in patients with sepsis, with the aim of generating hypotheses for future research.

## Materials and methods

### Methods

We conducted a retrospective propensity score-matched cohort study based on the Medical Information Mart for Intensive Care-IV (MIMIC-IV 3.1) database. The MIMIC-IV 3.1 database contains clinical information from hospitalized patients admitted to the adult intensive care units of Beth Israel Deaconess Medical Center between 2008 and 2022 (Johnson et al., 2023). The database has been approved by the Institutional Review Board of the Massachusetts Institute of Technology. One of our authors (Hua Xie) obtained a certificate authorizing access to the database after completing the required training (Record ID: 56163702). The preparation of this manuscript followed the Strengthening the Reporting of Observational Studies in Epidemiology (STROBE) statement (von Elm et al., 2007).

### Study population

This study included adult patients who met the Sepsis-3 diagnostic criteria. The diagnostic criterion was an increase in the Sequential Organ Failure Assessment score of ≥2 points due to a dysregulated host response to infection. For patients with multiple ICU admissions, only the first admission was included. Patients were excluded if they were younger than 18 years, had an ICU length of stay of less than 24 h, or had inaccurate time records in the database.

### Data extraction

Data extraction was performed using Structured Query Language. The SQL script code was obtained from the GitHub website (https://github.com/MIT-LCP/mimic-iv). We collected age, sex, and race, as well as data on disease severity [Sequential Organ Failure Assessment score, Glasgow Coma Scale score, Acute Physiology Score III, Charlson Comorbidity Index] and treatments received [invasive mechanical ventilation, vasopressor use, continuous renal replacement therapy]. We also collected comorbidities (myocardial infarction, congestive heart failure, cerebrovascular disease, hypertension, chronic pulmonary disease, liver disease, diabetes mellitus, renal disease, and malignancy), vital signs (heart rate, mean arterial pressure, respiratory rate, body temperature, and oxygen saturation), laboratory parameters [white blood cells, hemoglobin, platelets, creatinine, blood urea nitrogen, glucose, potassium, sodium, total calcium, bicarbonate, prothrombin time, international normalized ratio, partial thromboplastin time], as well as hospital length of stay and ICU length of stay.

### Exposure definition

Participants were divided into two groups based on whether they received carvedilol from the time of sepsis diagnosis to ICU discharge: the carvedilol group (intervention group) and the non-carvedilol group (control group).

### Outcome

The primary outcomes were 28-day and 365-day all-cause mortality, and the secondary outcome was in-hospital mortality.

### Statistical analysis

Continuous variables were presented as mean ± standard deviation or median (interquartile range), and categorical variables as frequency (percentage). For continuous variables, the Student’s t-test or Wilcoxon rank-sum test was used to assess normality. Categorical data were analyzed using the Pearson chi-square test or Fisher’s exact test, as appropriate.

Cox proportional hazards models were constructed to calculate hazard ratios with 95% confidence intervals for the primary outcomes (i.e., 28-day and 365-day all-cause mortality). The cumulative incidence of 28-day and 365-day all-cause mortality was analyzed using the Kaplan-Meier method and evaluated by the log-rank test. Logistic regression models were used to calculate odds ratios with 95% confidence intervals for the binary secondary outcome. The E-value was used to assess the impact of unmeasured confounding.

To balance baseline characteristics between the carvedilol and non-carvedilol groups, propensity score matching was performed using a 1:1 nearest-neighbor matching algorithm with a caliper width of 0.2. Variables included in the propensity score matching model were selected based on a published consensus statement (Austin, 2011) and included age, sex, race, Charlson Comorbidity Index, hypertension, diabetes mellitus, congestive heart failure, heart rate, mean arterial pressure, white blood cell count, total calcium, blood urea nitrogen, creatinine, international normalized ratio, Acute Physiology Score III, myocardial infarction, renal disease, malignancy, continuous renal replacement therapy, mechanical ventilation, and ICU length of stay. Subgroup analyses for the primary outcomes were performed based on sex (female vs. male), age (≤75 years vs. >75 years), Charlson Comorbidity Index (≤7 vs. >7), and renal disease (yes vs. no) (Wang et al., 2024). For covariates with missing values below 15%, multiple imputation was performed using random forest imputation, generating 10 complete datasets with 10 iterations per dataset (He, 2010).

In addition, sensitivity analyses were conducted: (1) exposure was treated as a time-dependent variable using a time-dependent Cox regression model to address potential immortal time bias; (2) the analysis was repeated after excluding patients with myocardial infarction, congestive heart failure, and hypertension to mitigate potential indication bias; (3) patients receiving other β-blockers (including metoprolol, bisoprolol, atenolol, propranolol, sotalol, labetalol, nebivolol, betaxolol, and esmolol) were used as the control group, and patients receiving carvedilol alone as the study group, to investigate whether the observed association was specific to carvedilol.

In all analyses, a two-sided P < 0.05 was considered statistically significant. Statistical analyses were performed using R software (version 4.2.3; R Foundation for Statistical Computing, Vienna, Austria).

## Results

### Basic characteristics

A total of 41,295 patients meeting the Sepsis-3 diagnostic criteria were extracted from the MIMIC-IV database. After excluding cases that met the exclusion criteria, 33,453 patients were finally included in the study. Among the study cohort, 1,084 patients received carvedilol after ICU admission, while the remaining 32,369 patients did not receive carvedilol (Figure 1).

**FIGURE 1:**
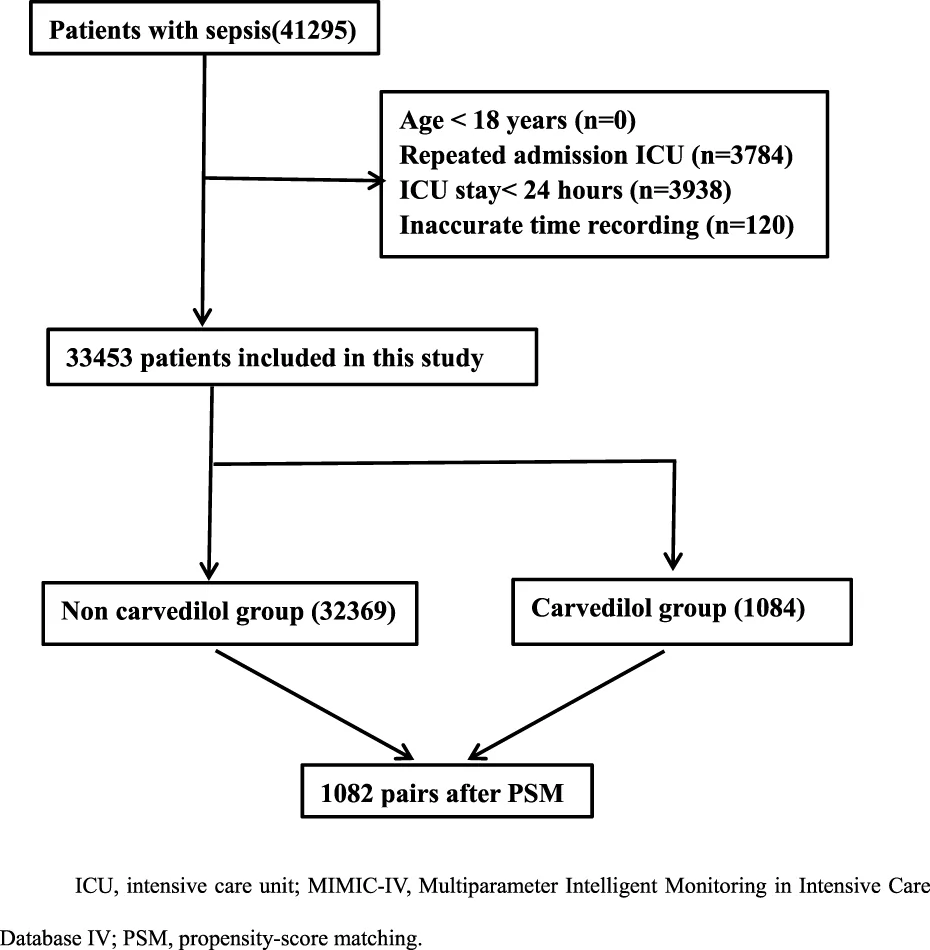
Flowchart of the patients screened in the study. ICU, intensive care unit; MIMIC-IV, Multiparameter Intelligent Monitoring in Intensive Care Database IV; PSM, propensity-score matching.

The baseline characteristics of patients before and after PSM are presented in Table 1. Compared with the control group, patients who received carvedilol were older (median [IQR], 70.51 [61.03, 78.83] vs. 67.89 [56.93, 78.56]; P < 0.001), had lower heart rate (median [IQR], 81.38 [72.22, 91.96] vs. 85.66 [75.60, 97.96]; P < 0.001), higher mean arterial pressure (median [IQR], 76.77 [71.06, 85.62] vs. 75.18 [69.64, 82.06]; P < 0.001), higher blood glucose (median [IQR], 143.00 [119.76, 182.22] vs. 132.36 [112.39, 163.60]; P < 0.001), and higher SOFA score (median [IQR], 5.00 [4.00, 8.00] vs. 5.00 [3.00, 7.00]; P = 0.001). The proportion of patients with myocardial infarction, congestive heart failure, cerebrovascular disease, diabetes mellitus, and renal disease was higher in the carvedilol group. After PSM, 1,082 patients who did not receive carvedilol were successfully matched with 1,082 patients who received carvedilol. Matching improved the balance of variables (SMD <0.1) (Table 1). Changes in SMD values for variables within the matching model before and after matching are shown in Figure 2.

**TABLE 1:** Baseline characteristics before and after propensity score matching.

Variable	Before propensity score matching				After propensity score matching			
	Non-carvedilol group (n = 32369)	Carvedilol group (n = 1084)	P	SMD	Non-carvedilol group (n = 1082)	Carvedilol group (n = 1082)	P	SMD
Age (median [IQR])	67.89 [56.93, 78.56]	70.51 [61.03, 78.83]	<0.001	0.172	70.73 [60.44, 80.41]	70.51 [61.04, 78.85]	0.573	0.013
Gender	13716/18653 (42.4/57.6)	392/692 (36.2/63.8)	<0.001	0.127	408/674 (37.7/62.3)	391/691 (36.1/63.9)	0.476	0.033
Male	18653 (57.6)	692 (63.8)	​	​	674 (62.3)	691 (63.9)	​	​
Female	13716 (42.4)	392 (36.2)			408 (37.7)	391 (36.1)		
Race	10918/21451 (33.7/66.3)	419/665 (38.7/61.3)	0.001	0.103	393/689 (36.3/63.7)	417/665 (38.5/61.5)	0.307	0.046
White	21451 (66.3)	665 (61.3)	​	​	689 (63.7)	665 (61.5)	​	​
Other	10918 (33.7)	419 (38.7)			393 (36.3)	417 (38.5)		
Los_hospital (median [IQR])	9.17 [5.59, 16.59]	10.73 [6.87, 18.82]	<0.001	0.098	9.88 [6.01, 17.34]	10.69 [6.87, 18.81]	0.01	0.028
Los_icu (median [IQR])	3.24 [1.88, 6.75]	4.44 [2.26, 9.02]	<0.001	0.189	3.46 [1.99, 7.76]	4.44 [2.26, 9.02]	<0.001	0.044
Vital signs								
Heart_ rate (median [IQR])	85.66 [75.60, 97.96]	81.38 [72.22, 91.96]	<0.001	0.278	81.31 [71.83, 93.27]	81.38 [72.25, 91.96]	0.936	0.001
MAP (median [IQR])	75.18 [69.64, 82.06]	76.77 [71.06, 85.62]	<0.001	0.236	77.36 [70.66, 85.56]	76.74 [71.05, 85.59]	0.881	0.008
Resp_ rate (median [IQR])	19.20 [16.73, 22.35]	19.22 [16.92, 21.91]	0.786	0.009	19.24 [16.95, 22.43]	19.22 [16.92, 21.90]	0.554	0.026
Temperature (median [IQR])	36.86 [36.58, 37.23]	36.86 [36.58, 37.20]	0.402	0.038	36.83 [36.57, 37.17]	36.86 [36.58, 37.20]	0.474	0.009
Spo2 (median [IQR])	97.26 [95.72, 98.62]	97.29 [95.57, 98.78]	0.494	0.028	97.35 [95.88, 98.65]	97.30 [95.57, 98.79]	0.544	0.038
Laboratory tests								
Glucose (median [IQR])	132.36 [112.39, 163.60]	143.00 [119.76, 182.22]	<0.001	0.029	137.00 [114.72, 172.95]	143.00 [119.70, 182.00]	0.003	0.037
Hemoglobin (median [IQR])	10.20 [8.87, 11.70]	10.06 [8.90, 11.58]	0.132	0.047	10.10 [8.90, 11.70]	10.07 [8.90, 11.59]	0.51	0.045
Platelets (median [IQR])	180.00 [126.75, 251.00]	178.83 [135.00, 239.00]	0.54	0.02	187.00 [133.50, 253.00]	178.83 [135.00, 239.00]	0.142	0.061
WBC (median [IQR])	11.70 [8.30, 15.94]	11.00 [8.12, 14.80]	<0.001	0.149	11.18 [8.07, 14.90]	11.00 [8.13, 14.80]	0.876	0.001
Bicarbonate (median [IQR])	23.00 [20.00, 25.50]	23.00 [20.67, 26.00]	<0.001	0.141	22.75 [20.00, 25.50]	23.00 [20.67, 26.00]	0.014	0.091
Bun (median [IQR])	22.33 [14.50, 38.00]	29.71 [18.50, 47.50]	<0.001	0.277	29.00 [18.00, 49.00]	29.67 [18.50, 47.25]	0.282	0.005
Calcium (median [IQR])	8.27 [7.80, 8.75]	8.47 [8.05, 8.90]	<0.001	0.286	8.44 [8.00, 8.90]	8.47 [8.05, 8.90]	0.181	0.055
Sodium (median [IQR])	138.50 [135.50, 141.00]	138.50 [136.00, 141.00]	0.702	0.014	138.55 [135.67, 141.46]	138.50 [136.00, 141.00]	0.669	0.011
Potassium (median [IQR])	4.18 [3.82, 4.60]	4.27 [3.90, 4.70]	<0.001	0.166	4.27 [3.89, 4.75]	4.26 [3.90, 4.70]	0.931	<0.001
Creatinine (median [IQR])	1.10 [0.77, 1.78]	1.43 [1.00, 2.50]	<0.001	0.315	1.40 [0.93, 2.45]	1.43 [1.00, 2.50]	0.094	0.021
INR (median [IQR])	1.30 [1.15, 1.57]	1.30 [1.15, 1.50]	0.119	0.023	1.30 [1.15, 1.60]	1.30 [1.15, 1.50]	0.181	0.052
PT (median [IQR])	14.35 [12.80, 17.10]	14.30 [12.60, 16.58]	0.066	0.044	14.30 [12.80, 17.40]	14.30 [12.60, 16.59]	0.124	0.066
PTT (median [IQR])	31.80 [27.84, 39.50]	32.30 [28.40, 41.30]	0.004	0.091	32.40 [28.08, 41.26]	32.30 [28.40, 41.28]	0.652	0.045
Severity of illness								
SOFA (median [IQR])	5.00 [3.00, 7.00]	5.00 [4.00, 8.00]	0.001	0.067	5.00 [4.00, 8.00]	5.00 [4.00, 8.00]	0.188	0.054
GCS (median [IQR])	15.00 [13.00, 15.00]	15.00 [14.00, 15.00]	0.116	0.032	15.00 [13.00, 15.00]	15.00 [14.00, 15.00]	0.694	0.006
APSIII (median [IQR])	47.00 [35.00, 63.00]	48.00 [37.00, 60.00]	0.556	0.016	48.00 [38.00, 61.00]	48.00 [37.00, 60.00]	0.525	0.02
Charlson comorbidity index (median [IQR])	5.00 [3.00, 7.00]	7.00 [5.00, 8.00]	<0.001	0.413	7.00 [5.00, 9.00]	7.00 [5.00, 8.00]	0.281	0.035
Comorbidity disease, n (%)								
Myocardial infarct	26667/5702 (82.4/17.6)	732/352 (67.5/32.5)	<0.001	0.348	725/357 (67.0/33.0)	732/350 (67.7/32.3)	0.783	0.014
No	26667 (82.4)	732 (67.5)	​	​	725 (67.0)	732 (67.7)	​	​
Yes	5702 (17.6)	352 (32.5)			357 (33.0)	350 (32.3)		
Congestive heart failure	22045/10324 (68.1/31.9)	359/725 (33.1/66.9)	<0.001	0.747	340/742 (31.4/68.6)	359/723 (33.2/66.8)	0.408	0.038
No	22045 (68.1)	359 (33.1)	​	​	340 (31.4)	359 (33.2)	​	​
Yes	10324 (31.9)	725 (66.9)			742 (68.6)	723 (66.8)		
Cerebrovascular disease	27533/4836 (85.1/14.9)	876/208 (80.8/19.2)	<0.001	0.113	895/187 (82.7/17.3)	874/208 (80.8/19.2)	0.266	0.05
No	27533 (85.1)	876 (80.8)	​	​	895 (82.7)	874 (80.8)	​	​
Yes	4836 (14.9)	208 (19.2)			187 (17.3)	208 (19.2)		
Chronic pulmonary disease	23519/8850 (72.7/27.3)	755/329 (69.6/30.4)	0.032	0.066	748/334 (69.1/30.9)	754/328 (69.7/30.3)	0.816	0.012
No	23519 (72.7)	755 (69.6)	​	​	748 (69.1)	754 (69.7)	​	​
Yes	8850 (27.3)	329 (30.4)			334 (30.9)	328 (30.3)		
Liver disease	27054/5315 (83.6/16.4)	957/127 (88.3/11.7)	<0.001	0.136	955/127 (88.3/11.7)	955/127 (88.3/11.7)	1.000	<0.001
No	27054 (83.6)	957 (88.3)	​	​	955 (88.3)	955 (88.3)	​	​
Yes	5315 (16.4)	127 (11.7)			127 (11.7)	127 (11.7)		
Diabetes	21887/10482 (67.6/32.4)	529/555 (48.8/51.2)	<0.001	0.389	540/542 (49.9/50.1)	529/553 (48.9/51.1)	0.667	0.02
No	21887 (67.6)	529 (48.8)	​	​	540 (49.9)	529 (48.9)	​	​
Yes	10482 (32.4)	555 (51.2)			542 (50.1)	553 (51.1)		
Renal disease	24171/8198 (74.7/25.3)	561/523 (51.8/48.2)	<0.001	0.489	547/535 (50.6/49.4)	561/521 (51.8/48.2)	0.576	0.026
No	24171 (74.7)	561 (51.8)	​	​	547 (50.6)	561 (51.8)	​	​
Yes	8198 (25.3)	523 (48.2)			535 (49.4)	521 (48.2)		
Malignant cancer	27872/4497 (86.1/13.9)	995/89 (91.8/8.2)	<0.001	0.182	982/100 (90.8/9.2)	993/89 (91.8/8.2)	0.446	0.036
No	27872 (86.1)	995 (91.8)	​	​	982 (90.8)	993 (91.8)	​	​
Yes	4497 (13.9)	89 (8.2)			100 (9.2)	89 (8.2)		
IMV	18074/14295 (55.8/44.2)	540/544 (49.8/50.2)	<0.001	0.121	547/535 (50.6/49.4)	539/543 (49.8/50.2)	0.763	0.015
No	18074 (55.8)	540 (49.8)	​	​	547 (50.6)	539 (49.8)	​	​
Yes	14295 (44.2)	544 (50.2)			535 (49.4)	543 (50.2)		
CRRT	30026/2343 (92.8/7.2)	987/97 (91.1/8.9)	0.038	0.063	1004/78 (92.8/7.2)	986/96 (91.1/8.9)	0.179	0.061
No	30026 (92.8)	987 (91.1)	​	​	1004 (92.8)	986 (91.1)	​	​
Yes	2343 (7.2)	97 (8.9)			78 (7.2)	96 (8.9)		
Vasoactive agent	17104/15265 (52.8/47.2)	597/487 (55.1/44.9)	0.156	0.045	567/515 (52.4/47.6)	595/487 (55.0/45.0)	0.244	0.052
No	17104 (52.8)	597 (55.1)	​	​	567 (52.4)	595 (55.0)	​	​
Yes	15265 (47.2)	487 (44.9)			515 (47.6)	487 (45.0)		
Hypertension	24674/7695 (76.2/23.8)	866/218 (79.9/20.1)	0.006	0.089	858/224 (79.3/20.7)	864/218 (79.9/20.1)	0.790	0.014
No	24674 (76.2)	866 (79.9)	​	​	858 (79.3)	864 (79.9)	​	​
Yes	7695 (23.8)	218 (20.1)			224 (20.7)	218 (20.1)		

IQR, interquartile range; MAP, mean arterial pressure; WBC, white blood cell; SOFA, sequential organ failure assessment; GCS, glasgow coma scale; APS III, acute physiology score; IMV, invasive mechanical ventilation; CRRT, continuous renal replacement therapy.

**FIGURE 2:**
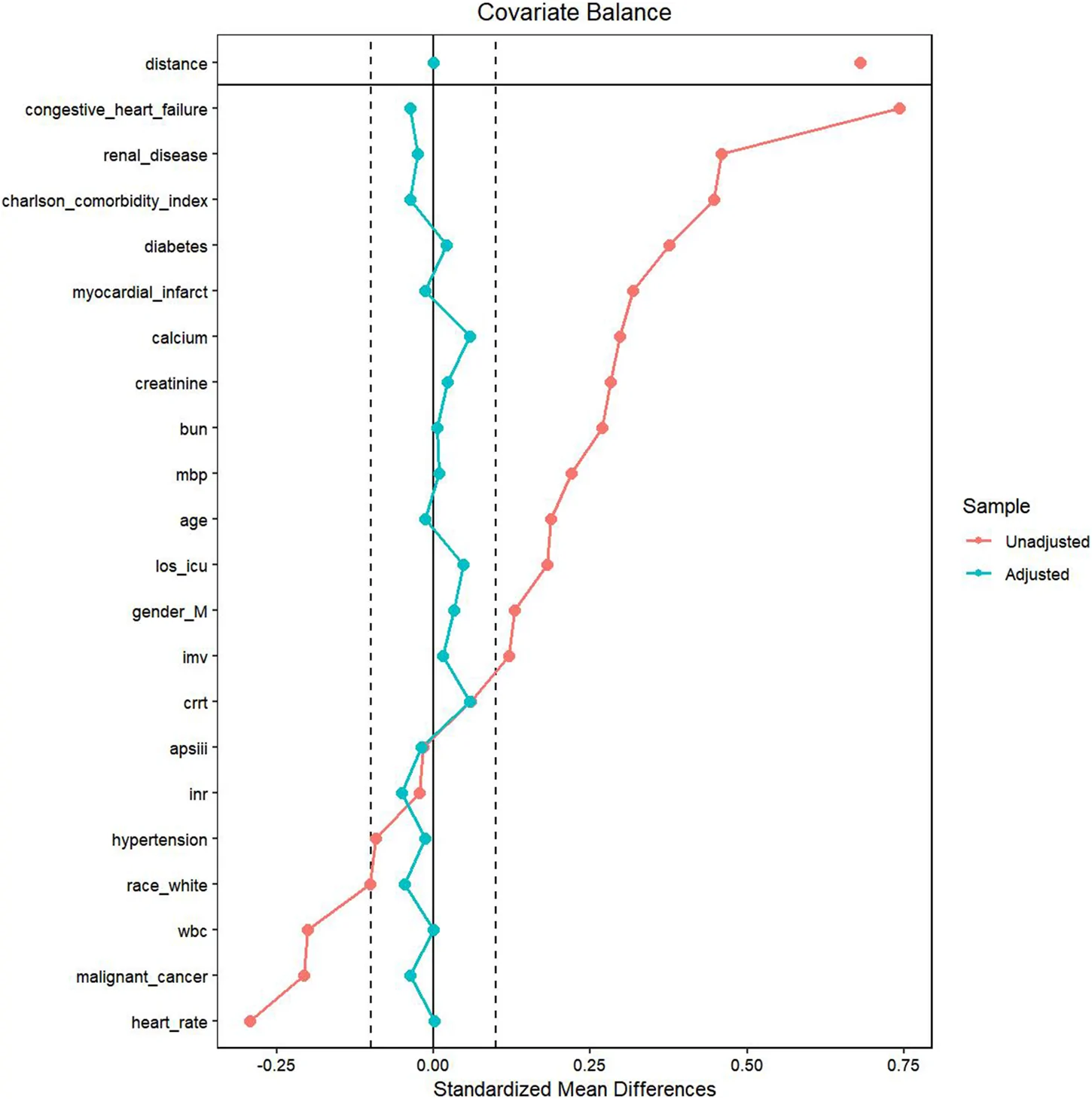
Changes in SMD values of variables within the matching model before and after matching.

### Primary outcomes

In the carvedilol group, the 28-day all-cause mortality rate was 12.1% (131/1082), compared with 21.2% (229/1082) in the non-carvedilol group. The 365-day all-cause mortality rate was 31.4% (340/1082) in the carvedilol group and 42.4% (459/1082) in the non-carvedilol group. Figures 3A,B show the Kaplan–Meier curves for 28-day and 365-day all-cause mortality according to carvedilol use in the matched cohort. Carvedilol use was associated with lower 28-day and 365-day all-cause mortality. In univariate Cox proportional hazards regression, the hazard ratio for 28-day all-cause mortality was 0.54 (95% CI, 0.43–0.67, P < 0.001), and the hazard ratio for 365-day all-cause mortality was 0.67 (95% CI, 0.58–0.77, P < 0.001). In multivariate Cox proportional hazards regression, the hazard ratio for 28-day all-cause mortality was 0.53 (95% CI, 0.43–0.66, P < 0.001), with an E-value of 3.18 (lower limit of 95% CI: 2.40); the hazard ratio for 365-day all-cause mortality was 0.66 (95% CI, 0.57–0.76, P < 0.001), with an E-value of 2.40 (lower limit of 95% CI: 1.96) (Table 2).

**FIGURE 3:**
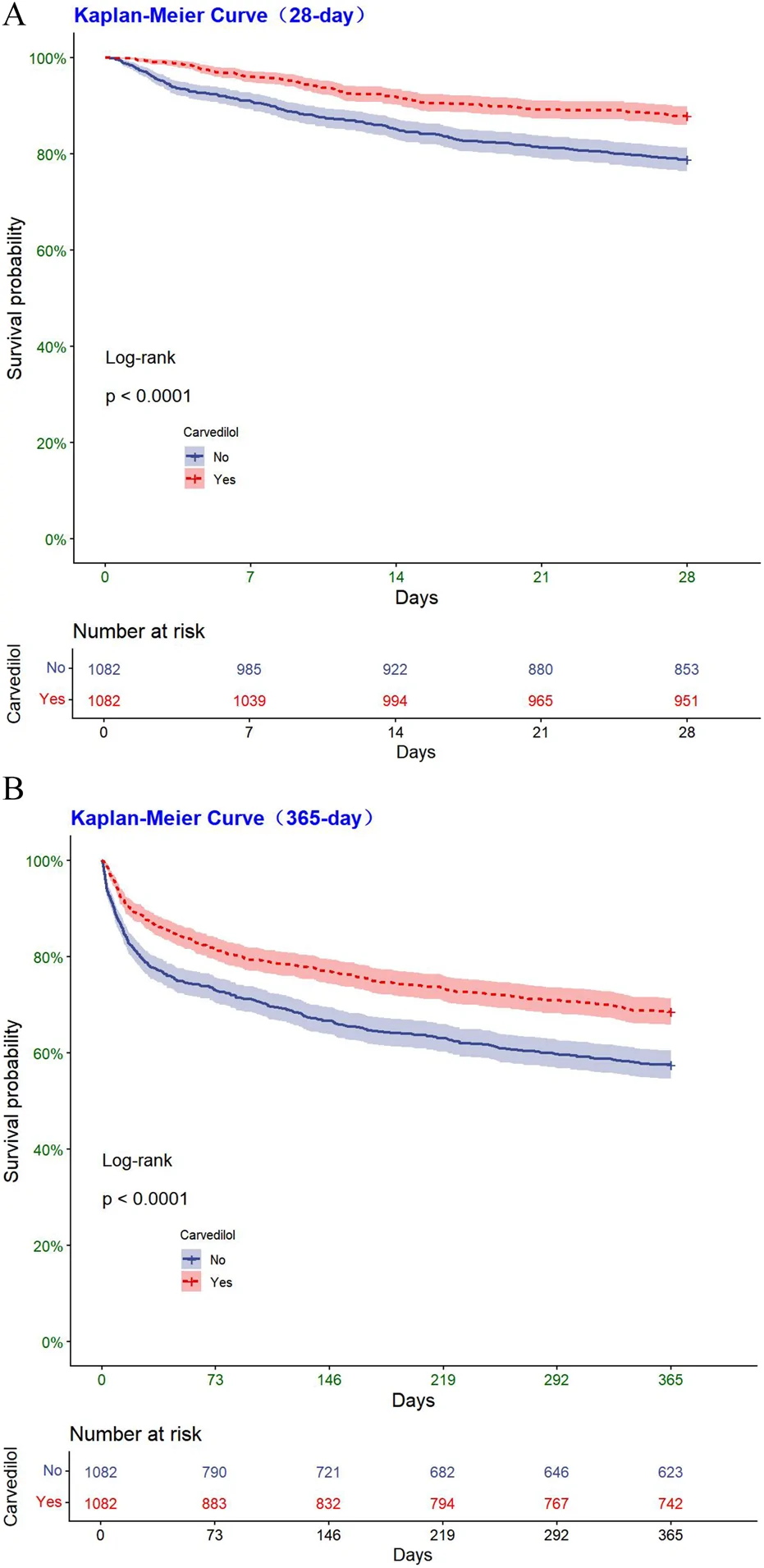
**(A)** Kaplan–Meier estimates for 28-day all-cause mortality according to carvedilol use in the matched cohort. **(B)** Kaplan–Meier estimates for 28-day all-cause mortality according to carvedilol use in the matched cohort.

**TABLE 2:** Association of carvedilol with primary and secondary outcomes.

Outcomes	Carvedilol group (*n* = 1082)	Non-carvedilol group (n = 1082)	Univariable analysis		Multivariable analysis		
			HR/OR (95% CI)	*P*-value	HR/OR (95% CI)	*P*-value	E-value (lower limit of the 95% CI)
Primary outcome							
28-day all-cause mortality†, *n* (%)	131 (12.1%)	229 (21.2%)	0.54 (0.43–0.67)	P < 0.001	0.53 (0.43–0.66)	P < 0.001	3.18 (2.40)
365-day all-cause mortality†, *n* (%)	340 (31.4%)	459 (42.4%)	0.67 (0.58–0.77)	P < 0.001	0.66 (0.57–0.76)	P < 0.001	2.40 (1.96)
Secondary outcomes							
In-hospital mortality‡, *n* (%)	104 (9.6%)	187 (17.3%)	0.51 (0.39–0.66)	P < 0.001	0.49 (0.37–0.64)	P < 0.001	3.50 (2.50)

† HR, with 95% CI, was calculated using Cox proportional hazards model.

* The adjusted covariates include age, gender, race, mean arterial pressure, glucose, international normalized ratio, Sequential Organ Failure Assessment (SOFA) score, Glasgow Coma Scale (GCS), Acute Physiology Score (APS) III, charlson comorbidity index, myocardial infarct, invasive mechanical ventilation, continuous renal replacement therapy, diabetes, hypertension.

### Secondary outcome

The in-hospital mortality rate was 9.6% (104/1082) in the carvedilol group and 17.3% (187/1082) in the non-carvedilol group. Carvedilol use was associated with lower in-hospital mortality in both univariate analysis (OR = 0.51; 95% CI: 0.39–0.66; P < 0.001) and multivariate analysis (OR = 0.49, 95% CI: 0.37–0.64; E-value: 3.50, lower limit of 95% CI: 2.50) (Table 2).

### Subgroup analysis

Figures 4A,B present the results of subgroup analyses for 28-day and 365-day all-cause mortality in the matched cohort. When the outcome was 28-day mortality, carvedilol use showed no significant interaction with any of the subgroups. When the outcome was 365-day mortality, carvedilol use showed no significant interaction with any subgroup except for the comorbidity index. In all other subgroups, the upper limit of the 95% confidence interval was below 1.00, indicating that carvedilol was associated with lower 28-day and 365-day all-cause mortality regardless of baseline patient characteristics.

**FIGURE 4:**
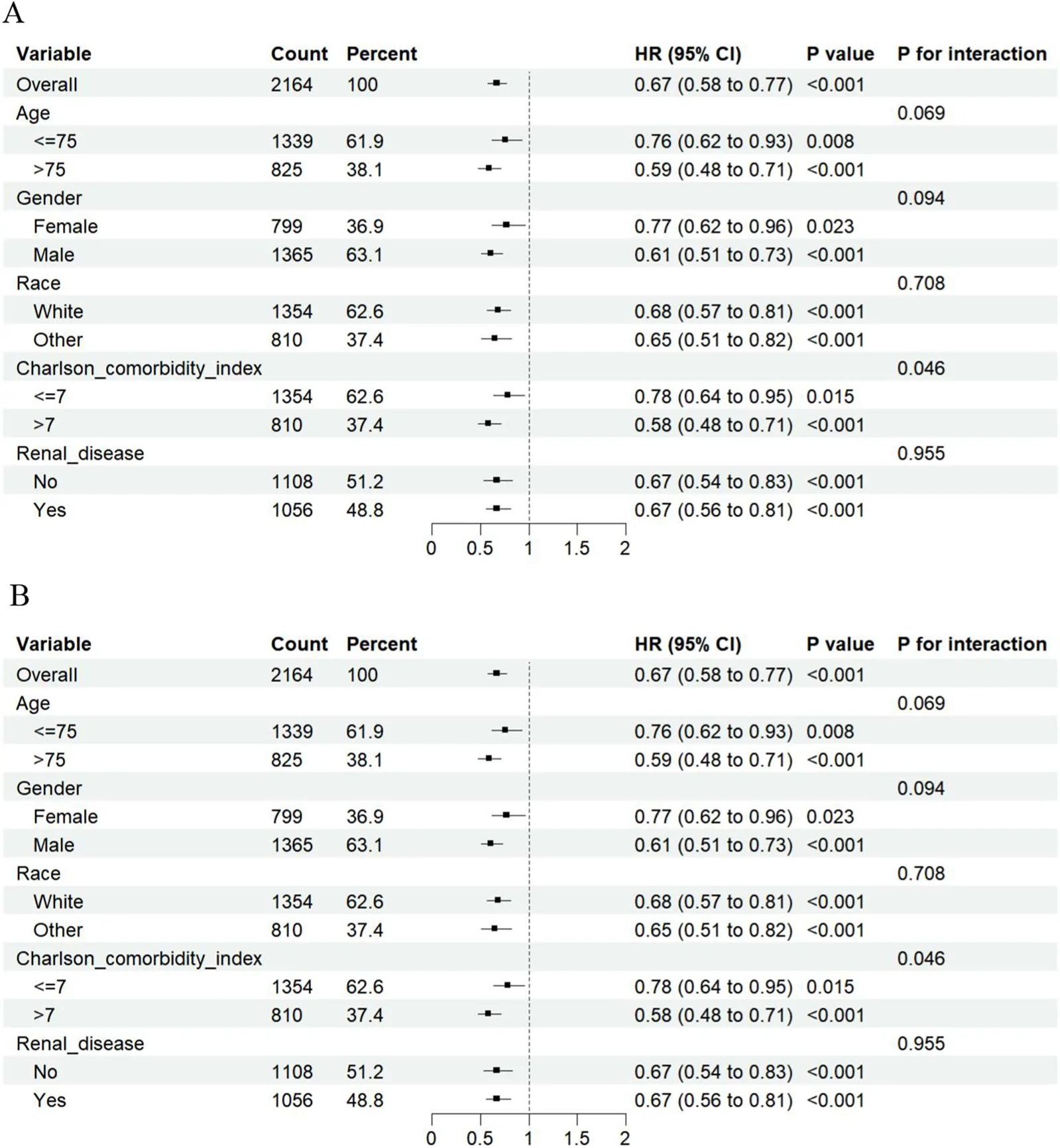
**(A)** Subgroup analyses for 28-day all-cause mortality in the matched cohort. **(B)** Subgroup analyses for 365-day all-cause mortality in the matched cohort.

### Sensitivity analysis

Whether using a time-dependent Cox model or excluding patients with myocardial infarction, congestive heart failure, and hypertension, carvedilol use remained significantly associated with reduced 28-day and 365-day mortality in patients with sepsis (Tables 3, 4). When patients receiving other β-blockers were used as the control group, carvedilol use was still associated with reduced 28-day mortality and in-hospital mortality in patients with sepsis, but there was no significant difference between the two groups for 365-day mortality (Table 5).

**TABLE 3:** Time-dependent Cox regression results for primary outcomes.

Outcomes	Univariable analysis		Multivariable analysis*	
	HR (95% CI)	*P*-value	HR (95% CI)	*P*-value
28-day all-cause mortality†, *n* (%)	0.74 (0.59–0.92)	0.006	0.75 (0.60–0.94)	0.01
365-day all-cause mortality†, *n* (%)	0.77 (0.67–0.89)	0.0002	0.76 (0.66–0.87)	0.0001

† HR, with 95% CI, was calculated using Cox proportional hazards model.

* The adjusted covariates include age, gender, race, mean arterial pressure, glucose, international normalized ratio, Sequential Organ Failure Assessment (SOFA) score, Glasgow Coma Scale (GCS), Acute Physiology Score (APS) III, charlson comorbidity index, myocardial infarct, invasive mechanical ventilation, continuous renal replacement therapy, diabetes, hypertension.

**TABLE 4:** Cox regression results after excluding patients with myocardial infarction, congestive heart failure, and hypertension.

Outcomes	Univariable analysis		Multivariable analysis*	
	HR (95% CI)	*P*-value	HR (95% CI)	*P*-value
28-day all-cause mortality†, *n* (%)	0.48 (0.32–0.73)	0.0007	0.41 (0.27–0.62)	P < 0.0001
365-day all-cause mortality†, *n* (%)	0.61 (0.46–0.81)	0.0005	0.50 (0.38–0.66)	P < 0.0001
In-hospital mortality‡, *n* (%)	0.38 (0.22–0.62)	0.0003	0.29 (0.16–0.49)	P < 0.0001

† HR, with 95% CI, was calculated using Cox proportional hazards model.

* The adjusted covariates include age, gender, race, mean arterial pressure, glucose, international normalized ratio, Sequential Organ Failure Assessment (SOFA) score, Glasgow Coma Scale (GCS), Acute Physiology Score (APS) III, charlson comorbidity index, invasive mechanical ventilation, continuous renal replacement therapy, diabetes.

**TABLE 5:** Cox regression results using other β-blockers as the control group.

Outcomes	Univariable analysis		Multivariable analysis*	
	HR (95% CI)	*P*-value	HR (95% CI)	*P*-value
28-day all-cause mortality†, *n* (%)	0.75 (0.57–0.98)	0.035	0.70 (0.54–0.92)	0.012
365-day all-cause mortality†, *n* (%)	0.98 (0.83–1.16)	0.84	0.86 (0.73–1.02)	0.078
In-hospital mortality‡, *n* (%)	0.57 (0.39–0.79)	0.001	0.53 (0.36–0.76)	0.0008

† HR, with 95% CI, was calculated using Cox proportional hazards model.

* The adjusted covariates include age, gender, race, mean arterial pressure, glucose, international normalized ratio, Sequential Organ Failure Assessment (SOFA) score, Glasgow Coma Scale (GCS), Acute Physiology Score (APS) III, charlson comorbidity index, myocardial infarct, invasive mechanical ventilation, continuous renal replacement therapy, diabetes, hypertension, malignancy cancer.

## Discussion

In this study, propensity score-matched analysis revealed that carvedilol use was independently associated with lower 28-day and 365-day all-cause mortality. This association remained consistent across all subgroup analyses and sensitivity analyses, indicating its robustness in our study cohort. Carvedilol use was also associated with lower in-hospital mortality.

### Discussion of the findings

First, in the analyses of the primary and secondary outcomes, the E-values were 3.18, 2.40, and 3.50, respectively (Table 2). The E-value represents the minimum strength of association that an unmeasured confounder would need to have with both the treatment and the outcome to fully explain the observed association. In this study, all E-values were greater than 2, indicating that the presence of strong unmeasured confounding sufficient to overturn the current conclusions is unlikely, thereby further supporting the robustness of the observed association.

Nevertheless, it should be borne in mind that this does not rule out the possibility of unmeasured confounding.

Second, subgroup analysis further revealed that the Charlson Comorbidity Index (CCI) acted as an effect modifier of the efficacy of carvedilol. Specifically, in patients with sepsis and a CCI >7, the association between carvedilol and lower 365-day mortality was more pronounced, suggesting that patients with a higher comorbidity burden may derive greater benefit (Figure 4B). Previous studies have also confirmed that CCI is an important effect modifier of β-blocker efficacy in sepsis, although they observed more pronounced benefits in patients with a CCI below the median (Wang et al., 2025). This discrepancy may be related to differences in study population characteristics–patients with a high CCI often have higher baseline sympathetic activity and inflammatory burden, thus leaving a larger margin of benefit for β-blockade.

Furthermore, this study found that carvedilol was superior to other β-blockers in reducing short-term mortality in patients with sepsis, but no significant difference was observed in long-term mortality. This may be explained by the following: the pleiotropic effects of carvedilol (antioxidant, anti-inflammatory, anti-apoptotic) play a critical protective role during the acute phase of sepsis, rapidly alleviating organ injury and reducing short-term mortality risk (Feuerstein et al., 1998; Drygała et al., 2024); other β-blockers (e.g., metoprolol, bisoprolol) lack these pleiotropic effects. Moreover, carvedilol may face challenges related to tolerability or adherence after the acute phase, whereas other β-blockers may gradually show benefits through sustained heart rate control and sympathetic inhibition during long-term follow-up, thereby producing a “catch-up effect” that diminishes the between-group difference in long-term mortality.

Finally, Of note, the exposure in this study was defined as carvedilol use during the period from sepsis diagnosis to ICU discharge, and our primary focus was to examine whether carvedilol use within this exposure window was associated with survival benefit, rather than to establish a causal effect between carvedilol therapy and outcomes. Future prospective data are warranted to guide the use of carvedilol in this population.

### Potential benefits of carvedilol in sepsis

The potential benefits of carvedilol in improving sepsis outcomes have received preliminary support from several studies. Okada et al. (Okada et al., 2016) used a cecal ligation and puncture-induced sepsis mouse model and found that carvedilol significantly reduced 7-day mortality. The underlying mechanism was related to activation of the “telomere-p53-PGC” signaling axis–carvedilol alleviated telomere shortening, upregulated PGC-1α expression, and promoted myocardial mitochondrial biogenesis, thereby ameliorating sepsis-induced cardiomyopathy. These findings provide translational evidence for the cardioprotective effects of carvedilol in sepsis.

At the clinical level, a large retrospective cohort study by Yang et al. (Yang et al., 2023) including 61,751 patients with sepsis showed that β-blocker use was associated with lower in-hospital mortality. Fuchs et al. (Fuchs et al., 2015) further distinguished different medication contexts: among patients newly initiated on oral β-blockers (including carvedilol) during sepsis, 90-day mortality decreased from 42% to 28%; whereas those who discontinued pre-existing β-blockers experienced an increase in mortality from 42% to 71%. Furthermore, a randomized controlled trial in patients with acute-on-chronic liver failure observed in secondary endpoints that the carvedilol group had significantly lower 28-day sepsis incidence (5% vs. 20%) and mortality (11% vs. 24%) compared with the placebo group. The median maximum tolerated dose was 12.5 mg (range 3.13–25 mg), consistent with routine clinical doses (Kumar et al., 2019).

### Comparison of carvedilol with other β-blockers: special considerations of α_1_-blocking properties

While interpreting the above associations, it is important to acknowledge the unique pharmacological properties that distinguish carvedilol from other β-blockers. Unlike selective β_1_-blockers such as esmolol and metoprolol, carvedilol is a non-selective β_1_/β_2_-blocker that also possesses α_1_-blocking activity, inducing vasodilation and reducing peripheral vascular resistance. This property is generally therapeutically beneficial in patients with hypertension or heart failure, but may pose a risk in patients with sepsis–a condition already characterized by vasoplegia due to systemic inflammation, excessive nitric oxide production, and vascular endothelial injury, leading to markedly increased sensitivity to vasodilatory effects (Burgdorff et al., 2018).

Currently, clinical evidence directly evaluating the hemodynamic safety of carvedilol in patients with sepsis is extremely limited. A study in an endotoxin-induced septic shock rat model (Kurita et al., 2009) showed that the high-dose carvedilol (10 mg/kg/day) pretreatment group had an 8-h mortality rate as high as 75%, compared with only 17% in the control group, and the treatment group exhibited more severe base deficit and elevated lactate, suggesting worsening tissue perfusion. It should be noted that the carvedilol dose used in that study was significantly higher than routine clinical doses (human equivalent dose approximately 1.6 mg/kg/day, whereas the usual clinical dose ranges from 0.05 to 0.4 mg/kg/day), and vasopressor support was not provided concurrently with carvedilol administration. Therefore, the carvedilol-associated benefit observed in our study should be primarily limited to hemodynamically stable patients with sepsis and cannot be extrapolated to patients with septic shock requiring high-dose vasopressors.

On the other hand, a large-scale propensity score-matched study based on the TriNetX database (5,166 patients per group) published in 2025 compared the effects of carvedilol and metoprolol in patients with left ventricular assist devices (Nwaezeapu et al., 2025). The results showed that the carvedilol group had significantly lower rates of sepsis incidence and all-cause mortality than the metoprolol group. Although the study population consisted of patients with left ventricular assist devices, these real-world data suggest that carvedilol may be associated with a lower risk of sepsis in patients with severe cardiovascular disease, an effect that may be related to its pleiotropic properties including antioxidant and anti-inflammatory actions.

### Mechanisms of potential benefit of carvedilol in sepsis

Synthesizing the available evidence, the biological plausibility of carvedilol improving sepsis outcomes can be explained from the following dimensions.

(i) Modulation of excessive sympathetic activation and tachycardia

During sepsis, the sympathetic nervous system is widely activated, with persistently elevated endogenous catecholamine concentrations that can lead to sustained tachycardia. Tachycardia has been established as an independent predictor of poor prognosis in patients with sepsis (Hayase et al., 2016). Increased heart rate shortens diastolic filling time, increases myocardial oxygen consumption, impairs coronary blood flow, and ultimately exacerbates myocardial depression. Carvedilol reduces heart rate through β_1_-receptor blockade, prolongs diastolic filling, and improves coronary perfusion. Consistent with the proven safety and efficacy of ultra-short-acting β_1_-blockers such as esmolol in patients with septic shock (Ruffolo et al., 1997), the heart rate-controlling effect of carvedilol may represent one of the bases for its benefit.

(ii) Cardioprotection and improvement of mitochondrial function

As noted above, carvedilol ameliorates cardiac contractile function in a mouse model of sepsis-induced cardiomyopathy by activating the telomere-p53-PGC signaling axis, alleviating telomere shortening, and promoting mitochondrial biogenesis (Okada et al., 2016). This mechanism distinguishes carvedilol from β-blockers that purely exert negative chronotropic or negative inotropic effects, suggesting that it may have unique organ-protective properties in the setting of sepsis-induced myocardial depression.

(iii) Anti-inflammatory and antioxidant effects

Unlike selective β_1_-blockers, carvedilol and its multiple metabolites possess additional anti-inflammatory and antioxidant properties (Feuerstein et al., 1998; Drygała et al., 2024). A 2023 study on sepsis-induced lung injury demonstrated that carvedilol significantly reduced the levels of pro-inflammatory cytokines such as TNF-α, IL-6, IL-1β, and HMGB1 (Karaali et al., 2023). *In vitro* studies have shown that carvedilol inhibits macrophage activity as well as hypoxia-induced NF-κB activation and reactive oxygen species generation (Gao et al., 2017). These anti-inflammatory and antioxidant effects may theoretically alleviate sepsis-related organ injury.

## Limitations

This study has several important limitations. First, the study used only the MIMIC database without external validation. Future multi-center cohort studies are needed to validate our findings. Second, due to the retrospective observational design, there may be unmeasured or excessively missing confounding factors, including serum lactate levels, vasopressor doses, fluid balance, source of infection, ventilator parameters, and other dynamic clinical severity indicators. Although we assessed the robustness of our findings using propensity score matching, sensitivity analyses, time-dependent models, and E-values, these approaches cannot completely eliminate the potential differences between carvedilol users and non-users that were not captured in the database—such as better hemodynamic stability or a more favorable baseline cardiovascular profile—which limits our ability to infer causality from the observed associations. Third, information on carvedilol exposure was incomplete. Information on dosage, timing of treatment initiation, duration of therapy, prior outpatient use, and whether treatment was interrupted during hospitalization was not fully available in the MIMIC-IV database, precluding more detailed exposure assessment and further causal inference. Future prospective data are needed to guide the use of carvedilol in this high-risk population.

## Conclusion

In conclusion, Carvedilol use during the period from sepsis diagnosis to ICU discharge was associated with reduced mortality in critically ill patients with sepsis. However, prospective studies are warranted to further guide the use of carvedilol in this population.

## Data Availability

The raw data supporting the conclusions of this article will be made available by the authors, without undue reservation.
